# The Post-Authorisation Maze: Mapping of European Pharmaceutical Market Access Pathways in the Early JCA Era

**DOI:** 10.3390/jmahp14030055

**Published:** 2026-09-17

**Authors:** Lisa-Maria Hagemann, Karolin Eberle, Jana Maurer, Alexandra Carls, Andreas D. Meid, Eva-Maria Reuter

**Affiliations:** *AMS* Advanced Medical Services GmbH, 80639 Munich, Germany; lisa-maria.hagemann@ams-europe.com (L.-M.H.); karolin.eberle@ams-europe.com (K.E.); jana.maurer@ams-europe.com (J.M.); alexandra.carls@ams-europe.com (A.C.); andreas.meid@ams-europe.com (A.D.M.)

**Keywords:** EU HTA regulation, joint clinical assessment (JCA), market access, oncology, innovative health technologies, European access environment, access timelines, health technology assessment, European economic area, EU big four

## Abstract

The Regulation (EU) 2021/2282 on health technology assessment (HTAR) harmonises clinical evidence assessment across Europe. However, national appraisal, pricing, and reimbursement processes remain divergent and could affect the efficiency of decision-making and patient access. Here we mapped the sequencing of national assessment, pricing, and reimbursement steps across 30 European countries. National pathways from marketing authorisation to reimbursed access were systematically identified and coded as categorical process sequences. Inpatient and outpatient pathways were analysed separately. Pathway dissimilarities were calculated using Optimal Matching and grouped by hierarchical clustering. We further explored whether clusters differed with regard to country-level indicators, including medicine availability, average time to access, gross domestic product, and healthcare expenditure. We found substantial procedural heterogeneity across countries in both inpatient and outpatient settings. Nevertheless, recurring pathway structures were identified. Across settings, the dominant distinction was between assessment-led pathways, in which evidence assessment precedes pricing and reimbursement steps, and pricing-led pathways, in which price formation occurs prior to assessment and reimbursement-related steps. Cluster membership was not associated with any country-level indicators. Our findings provide policymakers, agencies, and industry with a baseline for understanding national procedural heterogeneity and for evaluating how market access pathways evolve as the JCA is implemented.

## 1. Introduction

The time to patient access for new medicinal products varies widely across the European Union (EU) Member States [1]. The EU Pharmaceutical Strategy and the European Health Technology Assessment (HTA) Regulation (EU) 2021/2282 (HTAR) aim to accelerate access to innovative medicines by reducing duplication of work, thus improving the efficiency and predictability of market access processes across Europe [2,3]. A central instrument supporting this objective is the Joint Clinical Assessment (JCA), which has been implemented since January 2025 to harmonise the clinical evaluation of new medicines at EU level. While the content and methodology of clinical assessment are harmonised, national procedural steps related to appraisal, pricing, and reimbursement remain fully within Member State competence [2]. These procedural steps between marketing authorisation and reimbursement differ across countries, not only in terms of which steps are included, but also in their order, coordination, interactions, and which types of institutions are involved [4,5,6,7,8]. The organisation of HTA functions provides one example of this variation. Across the four largest EU markets, for instance, HTA functions are organised differently. Germany relies on an independent advisory body, the Institute for Quality and Efficiency in Health Care, which advises the Federal Joint Committee, whereas in France, the Transparency Committee and the Economic and Public Health Evaluation Committee are embedded within the Haute Autorité de Santé [4,5,6,7]. Italy represents a more integrated model, with the Scientific and Economic Committee for Medicines operating within the Italian Medicines Agency, while retaining technical-scientific autonomy [9]. Spain illustrates a more decentralised model, in which the 17 autonomous regions and hospital pharmacies may conduct HTA in addition to centralised assessment by the Spanish Agency for Medicines and Healthcare Products [10]. These structural and procedural differences may affect national decision-making and ultimately influence the timing and predictability of patient access.

Previous comparative research has also documented differences in reimbursement decisions and patient access to new medicines between EU Member States [1,11,12,13,14,15,16,17], identified HTA system archetypes and organisational features [7,8,10,18,19,20,21], and compared methodological approaches to HTA [15,22]. While this body of work has substantially improved understanding of national decision-making practices, comparatively little attention has been paid to the procedural sequencing of assessment, pricing, and reimbursement steps. Yet addressing this gap is particularly relevant in the context of the JCA, as differences in national processes may shape how jointly generated evidence is integrated into national decision-making, as recently noted by the European Federation of Pharmaceutical Industries and Associations (EFPIA) [23]. Evidence from cross-country analyses indicates that organisational and administrative factors can contribute substantially to differences in reimbursement timelines and access to new medicines [17].

We aim to identify patterns in European market access sequences including national assessment, pricing, and reimbursement steps using a data-driven analytic approach. We applied sequence and cluster analyses to group countries with similar pathway structures and explored whether identified clusters differed with regard to selected economic and access indicators. By shifting the analytical focus from institutional typologies and methodological differences to the sequencing of operational decision-making steps, this study adds a process-oriented perspective to the existing literature on national market access. We examine how appraisal, pricing, and reimbursement activities are ordered and coordinated within national pathways. In doing so, we establish a comprehensive baseline of market access pathways across all 30 European Economic Area countries participating in the JCA during its early implementation phase. This baseline provides a reference point for evaluating how national processes evolve over time in response to the introduction of the JCA and for identifying opportunities to improve the coordination and efficiency of post-JCA decision-making.

## 2. Methods

We analysed the procedural steps between marketing authorisation and reimbursed access, including all steps related to assessment, pricing, and reimbursement. We refer to the ordered sequence of these steps as a pathway. We focused on standard pathways for newly authorised medicinal products, primarily reflecting conventional non-orphan pricing and reimbursement processes. Alternative or non-standard pathways, such as orphan medicinal product procedures or pathways specific to products undergoing a joint clinical assessment, were not analysed separately unless they formed part of the standard national sequence. Inpatient and outpatient processes were analysed separately. For countries in which inpatient and outpatient pathways were identical, this was noted accordingly.

### 2.1. Data Collection

Data were collected systematically for each country according to a prespecified methodology. Sources included HTA agency process rules and guidance, national reimbursement legislation, and other publicly available country-specific documents, using the source framework described previously [24]. Additional information was obtained from official factsheets (e.g., [25,26]) as well as published literature such as book chapters and guides (e.g., [27,28,29,30]), and peer-reviewed journal articles describing processes in individual countries (e.g., [31,32,33,34,35,36]). Where no single formally defined or publicly documented pathway could be identified, for example because decision-making was decentralised, real-world practice was inferred using expert knowledge. Artificial intelligence tools (GPT-4o, Undermind, Perplexity) were used solely to support source identification and literature searching. No pathway information was derived directly from AI-generated content; all information included in the analysis was verified against original publications, official guidance, legislation, or other publicly available sources. Table 1 provides an overview of the key player and additional sources per country.

A categorisation framework was developed to enable a cross-country comparison of assessment, pricing and reimbursement process pathways in a step-by-step sequence from European Medicines Agency (EMA) marketing authorisation to reimbursed access. The framework systematically mapped process steps according to their presence and sequence. A process step was defined as a formally identifiable administrative or decision-making action performed by either the health technology developer (HTD) or a designated authority. Steps could occur more than once in a single sequence if the same action was performed by multiple distinct authorities. All possible steps considered across countries are listed in Table 2.

The number of steps could vary between countries, as not all countries can be expected to employ the complete set of process steps. Furthermore, the number of steps should not be interpreted as a proxy for overall process duration, as the analysis captures procedural sequencing rather than the time required to complete individual steps or the overall pathway. Where in practice steps occurred in parallel, they were represented sequentially in the dataset to allow consistent cross-country comparison, visualisation, and sequence analyses. For example, in Finland, Poland, and Hungary, price and reimbursement decisions were represented as sequential steps although they are made concurrently in practice. In the Danish inpatient pathway, price negotiation was represented after completion of the assessment, although negotiation may proceed in parallel with the assessment and decision-making process. In Italy, price negotiation and pricing and reimbursement decision-making may proceed iteratively and in parallel. In Belgium, assessment and price negotiation may also occur concurrently. Each country’s pathway was reviewed by at least two authors, and discrepancies were resolved by consensus.

### 2.2. Data Preparation and Statistical Analysis

Country-specific procedural pathways were represented as categorical sequences and analysed using sequence-analytic dissimilarity measures to quantify similarity between pathways. Inpatient and outpatient pathways were analysed separately. In countries with only one observed pathway for inpatient and outpatient products, that pathway was included in both the inpatient and outpatient analyses.

Each country pathway was represented as an ordered sequence of process steps. Distinct process steps were coded as unique sequence states. Sequences were retained at their observed lengths, with the final recorded process step treated as the pathway endpoint. Because the objective was to characterise procedural sequencing, pathways with three or fewer observed steps were excluded. This threshold was selected because very short sequences provide limited information for substitution- and indel-based distance estimation and may dominate similarity estimates through shared endpoints rather than meaningful procedural structure.

Sequence analyses were conducted in R (version 4.5.0) using the TraMineR package for sequence representation, distance computation, and visualisation [137]. Hierarchical clustering and silhouette diagnostics were performed using the packages ‘cluster’ and ‘mclust’. Dendrogram visualisation was supported by the ‘dendextend’ package.

Optimal Matching (OM) served as the primary sequence distance measure. Dissimilarity between pathways was calculated using transition rate-based substitution costs (TRATE), which assign lower costs to frequently observed transitions and higher costs to infrequently observed transitions. Insertions and deletions were assigned a constant cost of 1 [138,139]. This approach ensures that the distance metric reflects the empirically observed transition patterns. The resulting distance matrix was clustered using agglomerative hierarchical clustering with Ward’s minimum-variance method (Ward.D2) [140].

The number of clusters was determined using a data-driven approach based on the average silhouette width [140]. Candidate solutions ranging from k = 2 to k = 8 clusters were evaluated. The statistically optimal solution was defined as the one with the highest mean silhouette width. To favour interpretability and parsimony, the final solution was selected as the smallest number of clusters that both constituted a local maximum in the silhouette profile and reached at least 85% of the maximum average silhouette width. Silhouette diagnostics were further used to assess cluster quality and to identify observations with negative silhouette values.

A subsequent analysis aimed to identify whether the clusters in the sequence space were statistically different. Tests of sequence-space differences and cluster dispersion were conducted using the package ‘vegan’. In particular, differences in sequence space across clusters were assessed using permutational multivariate analysis of variance (PERMANOVA) [141]. Homogeneity of dispersion, a key assumption of PERMANOVA, was evaluated using betadisper based on OM distances [142]. For each cluster, a medoid sequence was identified to represent the most characteristic pathway within the cluster. The medoid was defined as the observed sequence with the smallest average dissimilarity to all other sequences in the same cluster, based on the OM distance matrix.

As an exploratory analysis, differences between clusters were further investigated using Kruskal–Wallis tests. Specifically, we tested whether the clusters differed with regard to pathway length (i.e., number of steps per country), percentage of drug availability [1], average time to access [1], GDP [143] and healthcare expenditure [144].

## 3. Results

Supplementary Figure S1 provides an overview of pathways for all 30 countries and both the inpatient and outpatient settings in alphabetical order as well as the average time to access for these countries.

### 3.1. Market Access Pathways for the Inpatient Setting

Of the 30 countries considered in the inpatient setting, we identified 26 unique sequence pathways. Austria, Latvia, and Liechtenstein were excluded from any further analysis as their pathways all consisted of only two steps. In all three cases, price negotiation is followed by reimbursed access (see Supplementary Figure S1). In practice, this means that prices are negotiated either by individual hospitals or by the government [33,41,42,88,90,91,145].

The remaining 27 countries were included in the cluster analysis for the inpatient setting.

**Figure 1:**
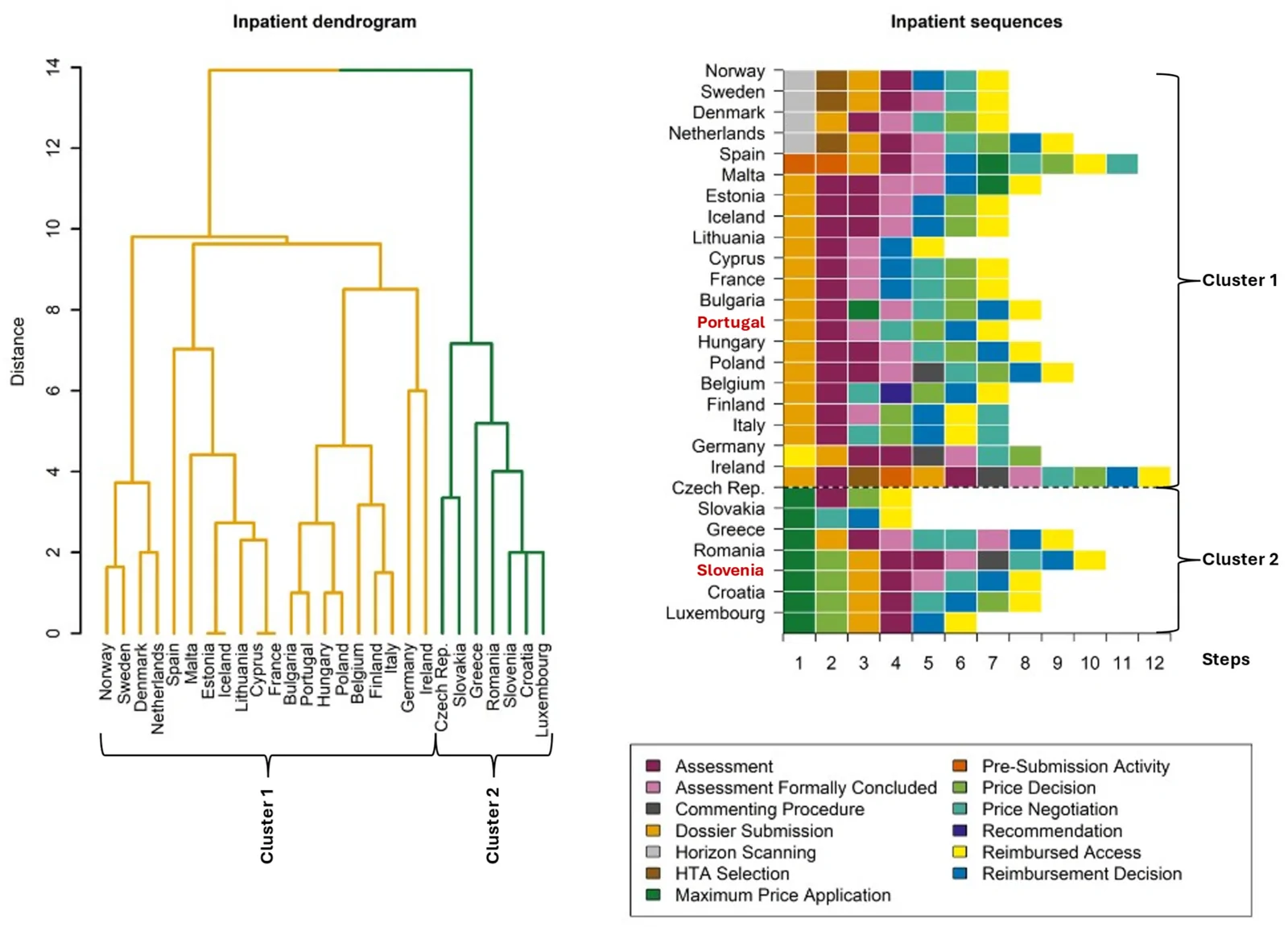
Dendrogram for pathways in the inpatient setting (**left panel**) and sequence plots grouped by cluster membership (**right panel**). Countries shown in red represent the most representative pathways of Clusters 1 and 2.

Based on the average silhouette width, a two-cluster solution was selected (see Supplementary Figures S2 and S3 for k selection and silhouette plots). Figure 1 presents the clustering results in the form of a dendrogram alongside the corresponding sequences. The two-cluster solution comprised 20 country pathways in Cluster 1 and seven country pathways in Cluster 2.

The average silhouette width (ASW = 0.268) indicated moderate cluster separation, with no negative silhouette widths. Thus, each sequence was, on average, more similar to sequences within its assigned cluster than to sequences in any other cluster, supporting the reliability of the cluster assignments. Consistent with this, the mean within-cluster distance (4.418) was lower than the mean between-cluster distance (5.956) and clusters differed significantly in sequence space (PERMANOVA: F(1,25) = 8.722, R^2^ = 0.259, *p* < 0.001), indicating that approximately 26% of the variation in sequence space was explained by cluster membership. Homogeneity of dispersion was supported by non-significant differences in within-cluster variability (betadisper ANOVA: F(1,25) = 0.346, *p* = 0.561). Clusters did not differ with regard to the pathway length (i.e., the number of steps included in each pathway; see Supplementary Table S1 for statistical results).

At the cluster level, distinct patterns emerged. Pathways in Cluster 1 generally follow an assessment-led structure with progression from dossier submission to assessment and then to pricing and reimbursement decisions (see Figure 1). Notably, this does not necessarily mean that dossier submission and assessment are the initial steps. In Denmark, the Netherlands, Norway and Sweden, for instance, the assessment is preceded by horizon scanning or HTA selection activities. Spain requires pre-submission activities, and in Germany reimbursed access is granted by default prior to any assessment. However, in all countries in Cluster 1, the assessment occurs early in the process and is followed by price-related steps. Based on the medoid sequence within each cluster, Portugal was identified as the most representative country for this cluster with a typical assessment-led pathway. In Portugal, the HTA dossier submission and assessment occur early and structure the subsequent process pathway, with pricing and reimbursement decisions following later [27,108,146].

Pathways in Cluster 2 follow a pricing-led structure, with an initial pricing-related step followed by subsequent assessment and formal decision-making steps, indicating a different ordering of key procedural components (see Figure 1). Based on the medoid sequence within this cluster, Slovenia was identified as the most representative country for the pricing-led type. In Slovenia, the market access process is initiated through an application to the national authority (Public Agency for Medicinal Products and Medical Devices of the Republic of Slovenia, JAZMP) responsible for determining the maximum allowed price (MAP) [147]. Dossier submission and clinical and economic assessment by the Health Insurance Institute of Slovenia (HIIS) occur subsequently as part of the reimbursement decision-making process [123,124].

### 3.2. Market Access Pathways for the Outpatient Setting

Of the 30 countries considered in the outpatient setting, we identified 28 unique sequence pathways. Liechtenstein was excluded as its process pathway consisted only of a single step: reimbursement decision. This decision is based on the Swiss assessment and reimbursement decision [90]. The remaining 29 countries were included in the cluster analysis.

**Figure 2:**
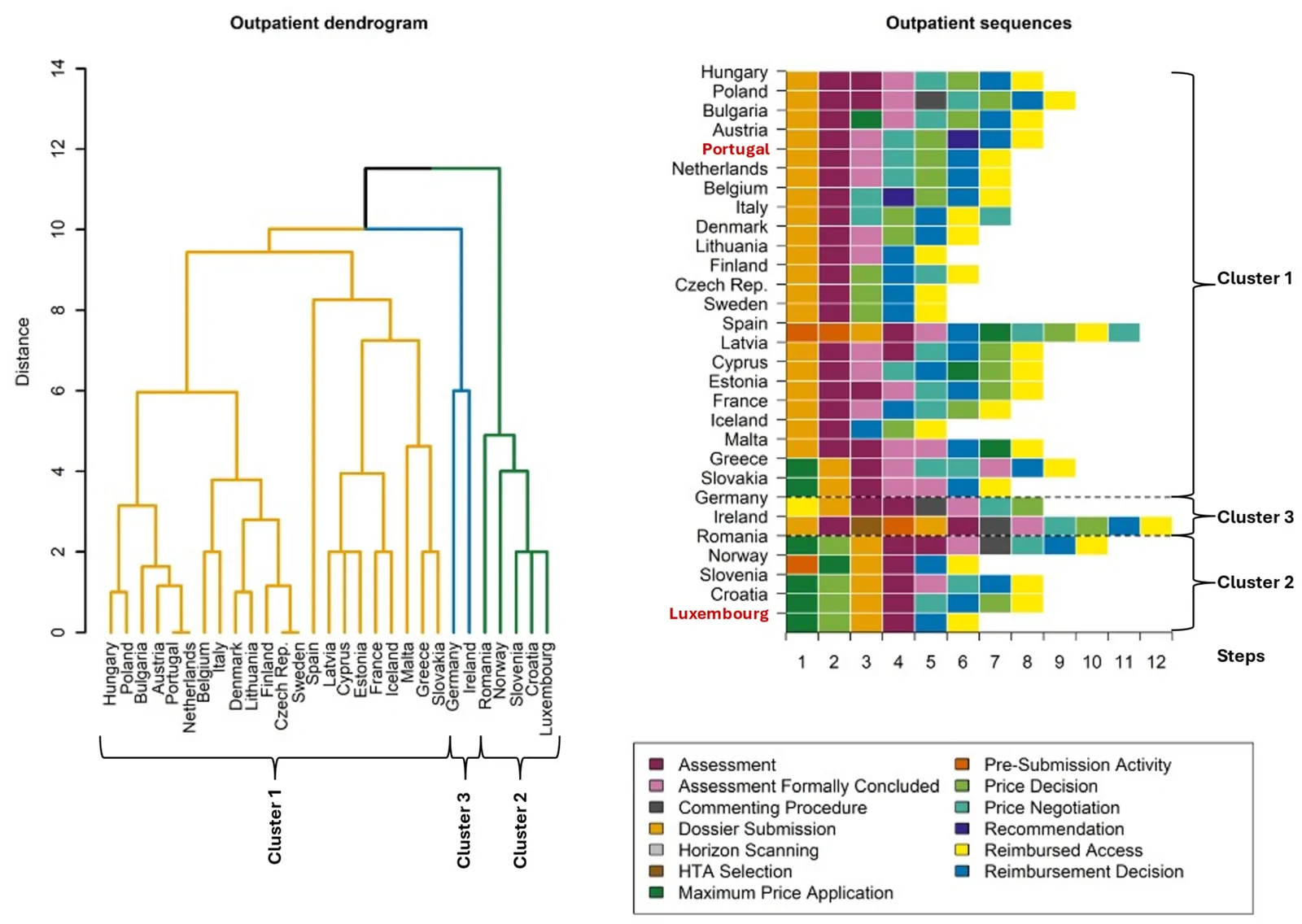
Dendrogram for pathways in the outpatient setting (**left panel**) and sequence plots grouped by cluster membership (**right panel**). Countries shown in red represent the most representative pathways of Clusters 1 and 2.

The average silhouette width supported a three-cluster solution (see Supplementary Figures S2 and S3 for k selection and silhouette plots), comprising 22 countries in Cluster 1, five countries in Cluster 2, and two countries in Cluster 3 (see Figure 2 for dendrograms and sequence plots).

Overall cluster validation metrics indicated moderate but consistent separation between clusters, comparable to the inpatient analysis (ASW = 0.258, mean within-cluster distance: 3.677, mean between-cluster distance: 5.630; PERMANOVA: F(2,26) = 7.113, R^2^ = 0.353, *p* < 0.001; betadisper ANOVA: F(2,26) = 0.541, *p* = 0.589). Again, cluster membership explained a significant proportion of variation in sequence configuration, while no evidence of unequal within-cluster dispersion was observed. Consistent with the inpatient setting, clusters did not differ with regard to the pathway length (i.e., number of steps included in each pathway; see Supplementary Table S1).

As in the inpatient setting, Cluster 1 includes countries with assessment-led pathways, with dossier submission and assessment preceding pricing and reimbursement decisions. Yet again this does not mean that these are the initial steps in all countries. In Greece and Slovakia, a maximum price application that is based on a reference price has to be made prior to the dossier submission and assessment, as this price is then included in the analysis presented in the dossier [31,75,117]. Again, Portugal is the most representative country for Cluster 1; its pathway is identical to the pathway in the inpatient setting.

Cluster 2 represents countries with pricing-led pathways. In all countries except Norway, the pathway starts with a maximum price application, followed by a price decision, dossier submission and assessment, formal decision-making, and reimbursed access. Norway follows the same pattern but requires a pre-submission activity. Luxembourg best represents this cluster. Its pathway starts with a maximum price application, followed by the price decision by the Ministry of Economy. Dossier submission and assessment are followed by the reimbursement decision, which results in the reimbursed access of the medicinal product [97].

Cluster 3 consists of only two countries, Germany and Ireland; therefore, we do not describe a medoid sequence. Their pairwise Optimal Matching dissimilarity was 6.0, compared with mean dissimilarities of 6.21 and 6.77 to countries assigned to Cluster 1 and 7.90 and 8.59 to countries assigned to Cluster 2 for Germany and Ireland, respectively. Thus, although Germany and Ireland were relatively closer to each other than to the other clusters, the magnitude of their mutual dissimilarity and the small cluster size provide limited evidence for a distinct and generalisable third pathway type. Cluster 3 was therefore interpreted as an atypical grouping rather than as a stable pathway archetype. In contrast to all other analysed countries, Germany is characterised by immediate reimbursed access following marketing authorisation, meaning that medicinal products with a valid EMA marketing authorisation and listing in the German pharmaceutical pricing database are automatically reimbursed upon launch. Hence, a distinct reimbursement decision step is omitted in Germany, contrasting with all other countries included in the analysis [148]. Ireland, the second member of this cluster, is characterised by an initial dossier submission followed by a rapid review to determine whether a full HTA is required. If a full HTA is deemed necessary, a comprehensive dossier is submitted for evaluation by the National Centre for Pharmacoeconomics (NCPE). Pricing and reimbursement decisions are then made upon completion of the assessment [27,36,149]. Despite these procedural differences, both pathways share more characteristics with an assessment-led than with a pricing-led logic, which is also reflected in the relatively smaller mean dissimilarities to countries assigned to Cluster 1 than Cluster 2. As both Germany and Ireland have identical pathways for the inpatient and outpatient settings, this interpretation is consistent with their assignment to the assessment-led inpatient Cluster 1.

Overall, the outpatient analysis confirms the presence of the same underlying logics observed in the inpatient setting. Because Cluster 3 comprised only two countries, we interpret it cautiously as an atypical pathway group rather than a stable third pathway type.

### 3.3. Empirical Links Between Clusters and Country-Level Markers

We explored whether clusters differed with regard to country-level markers, specifically medicinal product availability and time to access, as well as GDP and health expenditure. We did not detect any differences between clusters in these exploratory analyses (Supplementary Figure S1 shows average time to access alongside the inpatient and outpatient sequences per country in alphabetical order; Supplementary Figures S4 and S5 show box plots for all variables; Supplementary Table S1 provides statistical results). Given the small number of countries and unbalanced cluster sizes, these findings should be interpreted cautiously.

## 4. Discussion

This study systematically mapped national assessment, pricing, and reimbursement pathways from centralised marketing authorisation to reimbursed access across all 30 European countries that take part in the JCA. We show substantial procedural heterogeneity between countries in both the inpatient and the outpatient settings, with the vast majority of countries exhibiting unique sequence pathways. Despite this heterogeneity, sequence analysis identified recurring pathway types across countries and settings. The main distinction was between assessment-led pathways and pricing-led pathways. These findings imply that, despite increasing harmonisation of clinical evidence assessment at the European level, national systems are likely to remain heterogeneous with distinct national procedural logics.

Previous research has shown considerable variation between European HTA and reimbursement systems with respect to institutional design, HTA archetypes, assessment methods, decision criteria, and access timelines [1,4,7,8,18,19,20,21]. This study adds a complementary perspective by showing that variability between European countries also extends to the temporal and procedural organisation of market access pathways. Our results imply that the identified assessment-led and pricing-led pathway types represent more than differences in procedural order. In assessment-led systems, clinical and/or economic evidence assessment is the central organising element of the pathway, with pricing and reimbursement decisions generally depending on the outcome of the assessment. By contrast, pricing-led systems initiate price formation or maximum price setting at an earlier stage, making these a prerequisite for subsequent assessment and reimbursement decisions. Because clusters were derived from the overall sequencing of steps across the entire pathway, we suggest that observed procedural differences between pathway types may reflect broader system logics and, potentially, socio-cultural preferences regarding how evidence assessment, affordability considerations, and reimbursement decisions are integrated within national healthcare systems.

We further explored whether pathway clusters differed in selected country-level characteristics or access indicators, including GDP, healthcare expenditure, medicine availability, and average time to access, but did not observe any significant differences between clusters. Given the small number of countries and unbalanced cluster sizes, these exploratory findings should be interpreted cautiously and should not be considered evidence that sequencing and market access outcomes are unrelated. Rather, they suggest that pathway type alone does not explain cross-country differences in access indicators. This interpretation is consistent with previous work showing that HTA and reimbursement systems cannot be reduced to geography or economic capacity alone, but are also shaped by institutional design, decision criteria, and value-based considerations [5,7,22,150,151]. However, the lack of an observed association with average time to access may appear counterintuitive, given that our study focuses on the procedural organisation of market access pathways. Importantly, however, our analysis captures the order of procedural steps, not the duration of individual steps or the intervals between them. Previous work has similarly shown that access timelines are influenced by multiple factors, including financial, organisational, administrative, and capacity-related factors [17]. Additional factors not directly encoded in sequence structure include manufacturer launch strategies, dossier completeness and related requests for further evidence or analyses, agency capacity, statutory or non-statutory review timelines, and the duration of price negotiations. Procedural sequencing should therefore be understood as one component of the broader market access ecosystem, rather than as a direct proxy for speed of access.

Similarly, pathway sequencing cannot be equated with submission-related workload. Such workload cannot be inferred from publicly available dossier templates or procedural descriptions alone [150]. It is a multidimensional construct that is affected by the volume of documentation required, the complexity of evidence generation (e.g., indirect treatment comparisons), methodological heterogeneity across jurisdictions, requests for additional or locally contextualised analyses, payer requirements, and the intensity of interactions with national authorities. In addition, HTA bodies may request further analyses after submission or reject dossiers that do not meet minimum quality requirements, indicating that dossier preparation can be an iterative process [152,153].

### 4.1. National Market Access Routes in the JCA Era

Our data describe national pathways prior to any JCA-associated changes. Conceivably, pathways might change in the future. However, our current findings suggest that national decision-making processes are likely to remain distinct despite the harmonisation of clinical evidence assessment under the EU HTAR. This may be particularly relevant for orphan medicinal products, which will enter the scope of the JCA from 2028. Evidence generation in rare diseases may be constrained by small patient populations and limited availability of effective comparators, which may increase the use of single-arm trials, real-world evidence, or external control data [154]. A recent study suggests that single-arm evidence may pose challenges for establishing relative effectiveness within the JCA framework, although such evidence may still inform national HTA decision-making [155]. Thus, while JCA could reduce duplication by providing a common clinical assessment of the available evidence, differences in how JCA outputs and residual evidence uncertainty are incorporated into national decision-making may contribute to continued variation in access to orphan medicinal products across Europe.

The extent to which JCA can contribute to faster and more predictable market access will not only depend on the quality of the shared assessment reports but also on how national processes integrate the JCA outputs into existing decision-making frameworks [156,157,158]. Differences in sequencing and procedural arrangements may introduce additional coordination requirements, administrative dependencies, or waiting periods that influence how decision-making procedures are organised and managed [5,159]. Early triaging procedures in some countries, such as Ireland’s NCPE “Rapid Review,” may experience delays under the HTAR non-duplication rule [158]. The heterogeneity of HTA across countries, particularly regarding clinical value assessment and pharmacoeconomic evaluation, implies that the extent and timing of integrating JCA outputs into national systems may vary significantly [157]. The availability of high-quality comparative effectiveness evidence can, however, reduce the need for full HTAs, and the utility of the JCA depends on whether its results can be incorporated into treatment-effectiveness inputs for cost-effectiveness modelling [158,160].

Nevertheless, national procedural features such as submission windows, horizon scanning, rebuttal or commenting procedures, price negotiation, and national requirements for economic evaluation are likely to remain important determinants of access timelines following JCA implementation. Accordingly, potential opportunities to maximise the benefits of the JCA include synchronising national submission timelines with the availability of JCA reports, clarifying when and how additional national contextual evidence should be submitted, increasing transparency around decision gates and commenting procedures, and encouraging parallel handling of assessment, pricing, and reimbursement steps where feasible. Such adjustments could help Member States benefit more from the JCA while maintaining national competence over pricing and reimbursement. Several countries have already begun adapting their national HTA requirements and processes to align with the JCA. For example, Germany and Belgium have revised their submission templates to avoid requesting clinical evidence that is already covered by the JCA [161,162]. Similarly, Latvia and Portugal have integrated the JCA report into their national HTA processes [109,163]. Other countries have used the implementation of the JCA as an opportunity for broader institutional reform: Slovakia and Luxembourg have established new HTA institutions [98,118], Spain is in the process of setting up a new system [130], while Austria is adapting its assessment processes to accommodate the JCA [43].

National implementation of the JCA is still evolving, and practical experience remains limited. As of September 2026, only four JCA reports have been published. It is therefore not yet possible to consistently determine from publicly available sources whether individual national process steps will be initiated before, proceed in parallel with, or depend on the JCA report. Future research should therefore map these interfaces once greater practical experience has accumulated and national procedures are more clearly established. Ultimately, the extent to which national adaptations will translate into faster and more predictable patient access remains to be evaluated in the future. Our study provides a baseline for future analysis of how national assessment, pricing, and reimbursement pathways evolve following JCA implementation and whether the procedural adaptations contribute to measurable changes in access timelines.

### 4.2. Methodological Considerations

Several methodological aspects should be considered. First, the analysis represents formal or publicly documented pathways. In some countries, especially in the inpatient setting, processes are decentralised, less clearly documented, or partly shaped by real-world institutional practice. In such cases, pathway steps had to be inferred from sparse available sources and expert know-how, which may not fully capture local operational complexity. Second, because parallel activities had to be represented sequentially for the sequence analysis, our approach does not capture the degree of temporal overlap between procedural steps. This may be particularly relevant for JCA implementation, as the ability to conduct national activities in parallel with, or immediately following, the JCA could influence overall process efficiency, although our analysis does not allow us to determine whether parallel processing itself leads to greater efficiency. Third, cluster separation was moderate, indicating that countries may not form sharply distinct procedural models but instead occupy overlapping positions along a spectrum of pathway configurations or represent highly specific organisational models. This limitation is also reflected in the outpatient analysis, where one cluster comprised only Germany and Ireland. Given its small size, this cluster should be interpreted cautiously as an atypical grouping rather than as evidence of a stable third pathway type. Fourth, although inpatient and outpatient pathways were analysed separately, they were not fully independent because some countries had identical pathways across both settings, whereas others had distinct setting-specific pathways. A systematic comparison between inpatient and outpatient settings would be a relevant direction for future research.

### 4.3. Practical Recommendations and Outlook

This study provides a baseline of European market access pathways in the early JCA era, including 30 European countries. We show that national sequence pathways are highly heterogeneous but follow two main sequencing logics: assessment-led pathways and pricing-led pathways.

These findings are relevant for several stakeholder groups. For policymakers and HTA bodies, they highlight heterogeneity and suggest the need to align national procedures with JCA outputs where possible. For manufacturers, understanding whether a system follows an assessment-led or pricing-led logic may support more targeted launch planning, dossier timing, and resource allocation. In assessment-led systems, early alignment with clinical and economic evidence requirements may be critical. Notably, Germany, France, Spain, and Italy, the four largest EU markets, were assigned to the assessment-led type. Efficient integration of JCA outputs into these national assessment procedures may have a substantial impact on reducing duplication and accelerating patient access across Europe. In pricing-led systems that we identified in some Eastern European countries but also in Luxembourg, manufacturers may need to prepare earlier for price-related submissions, maximum price applications, and negotiation strategies before or alongside evidence assessment. Recognising these sequencing logics might help to anticipate procedural dependencies, allocate resources across markets more efficiently, and better coordinate European JCA timelines with national submission and access strategies.

As the JCA does not apply in the UK, the UK was beyond the scope of the present analysis. However, future research could explore potential interactions between NICE requirements, JCA-related evidence generation, and national market access processes across Europe. To date, the JCA has begun to shape national assessment, methodological requirements, and institutional reforms [98,109,118,130,161,162,163], but has not yet had a substantial impact on national procedural pathways. Dedicated pathways for products falling under the EU HTA Regulation remain uncommon [43]. Even where JCA-specific procedural adaptations are introduced, national sequencing logics are likely to remain important in determining how marketing authorisation and JCA outputs translate into reimbursed patient access. We therefore recommend close monitoring of the evolving national market access processes and evidence requirements, particularly when planning European regulatory and launch sequences. Future research should assess whether countries adapt their processes in response to the EU HTA Regulation, whether the identified pathway types remain stable, and whether procedural changes translate into faster and more predictable patient access. Future research could map the specific interfaces between the JCA and national market access pathways, including which national activities can be initiated before availability of the JCA report, which can proceed in parallel, and where procedural dependencies or potential bottlenecks arise. In this context, combining a step-based characterisation of national procedures with a systematic assessment of submission-related burden could provide valuable insights into whether the JCA reduces duplication, redistributes workload, or creates new coordination requirements across national processes. Such an integrated approach may help to identify whether, and in which countries, the JCA has the potential to reduce duplication of effort and streamline evidence requirements.

## Figures and Tables

**Table 1:** Key national players involved in assessment, pricing and reimbursement procedures across the 30 countries included in the analysis.

Member State	Key National Players	Further Readings
Austria	The Austrian social insurance funds [37] Medicinal Products Evaluation Commission [38]	[33,39,40,41,42,43]
Belgium	National Institute for Health and Insurance (NIHDI-INAMI-RIZIV) [44]	
Bulgaria	National Council on Prices and Reimbursement of Medicinal Products [45] National Health Insurance Fund [46]	[11,47,48]
Croatia	Agency for Medicinal Products and Medical Devices (HALMED) [49] Croatian Health Insurance Fund [50]	[30]
Cyprus	Health Insurance Organisation [51] Ministry of Health [52]	[30,53,54]
Czech Republic	State Institute for Drug Control [55]	
Denmark	Danish Medicines Agency [56] Danish Medicines Council (DMC) [57]	
Estonia	Estonian Health Insurance Fund [58] State Agency of Medicines [59] Ministry of Social Affairs [60]	
Finland	Pharmaceuticals Pricing Board [61] Finnish Medicines Agency (Fimea) [62] Council for Choices in Health Care in Finland [63] Finnish Coordinating Center for Health Technology Assessment (FinCCHTA) [64]	
France	French National Authority for Health (HAS) [65] Ministries of Health & Social Security [66] Economic Committee for Health Products (CEPS) [67] National Union of Health Insurance Funds (UNCAM) [68] French National Agency for Safety of Medicines (ANSM) [69]	[35,70]
Germany	Federal Joint Committee (GBA) [71] Institute for Quality and Efficiency in Health Care (IQWiG) [72] GKV-Spitzenverband [73]	
Greece	Ministry of Health [52]	[31,74,75]
Hungary	National Health Insurance Fund of Hungary [76]	[11,77,78]
Iceland	Icelandic Medicines Agency [79] The National University Hospital of Iceland [80]	
Ireland	Health Service Executive [81] National Centre for Pharmacoeconomics (NCPE) [82]	[27,36,83]
Italy	Italian Medicines Agency (AIFA) [84]	
Latvia	National Health Service [85] State Agency of Medicines [86]	[87,88]
Liechtenstein	Office of Public Health [89]	[90,91]
Lithuania	State Medicines Control Agency [92] National Health Insurance Fund [93] Ministry of Health [94]	
Luxembourg	National Health Fund [95] Ministry of economy [96]	[97,98]
Malta	Ministry for Health [99]	[32,100,101]
Norway	Norwegian Medical Products Agency (DMP/NOMA) [102] Regional Health Authorities [103] Norwegian Hospital Procurement Trust [104]	
Poland	Agency for Health Technology Assessment and Tariff System (AOTMiT) [105] Ministry of Health [106]	
Portugal	National Authority of Medicines and Health Products (INFARMED) [107]	[108,109]
Romania	National Agency for Medicines and Medical Devices [110] Ministry of Health [111] National Health Insurance House [112]	[113]
Slovakia	National Institute for Value and Technology in Healthcare (NIHO) [114] Ministry of Health [115]	[30,116,117,118]
Slovenia	Health Insurance Institute of Slovenia [119] Agency for Medicinal Products and Medical Devices of the Republic of Slovenia (JAZMP) [120]	[121,122,123,124]
Spain	Spanish Agency of Medicines and Medical Devices (AEMPS) [125] Ministry of Health [126] Interministerial Committee on the Price of Medicines [127]	[128,129] [10,130]
Sweden	Dental and Pharmaceutical Benefits Agency (TLV) [131] Regional Medicines Collaboration Model [132]	
The Netherlands	National Health Care Institute (ZIN) [133] Ministry of Health, Welfare and Sport [134]	[135,136]

**Table 2:** Definition of the procedural steps of national assessment, pricing, and reimbursement.

Procedural Steps	Description
Assessment	Evaluation of the submitted evidence by one or more institutions or authorities. When multiple authorities performed separate assessments, distinct process steps were created to reflect institutional responsibilities (e.g., separation of clinical and economic evaluations). When multiple assessments were conducted within the same authority, they were consolidated into a single step. Formal completeness checks and other administrative validation activities preceding the actual assessment were integrated into the assessment step when performed by the same authority. However, where assessment activities are distributed across multiple stakeholders or procedural stages, they were retained as separate steps to reflect process complexity.
Assessment formally concluded	To ensure consistent representation of assessment outcomes across countries, a standardised step termed “Assessment formally concluded” was introduced. This step reflects the completion of an evaluation process in all cases where an assessment of the submitted data takes place, regardless of whether a formal HTA decision is issued. Across countries, the conclusion of the assessment phase may be communicated through a formal decision, notification to the manufacturer, or implicitly through a recommendation to the next authority. The step “Assessment formally concluded” was therefore used as a common functional endpoint of the evaluation phase and also allows inclusion of countries where no formal HTA process exists but where an evaluation is nevertheless conducted.
Commenting Procedure	Formal opportunity for stakeholders (e.g., HTD or external experts) to review and comment on draft assessment reports or preliminary decisions prior to finalisation.
Dossier Submission	Submission of a formal dossier or application by the HTD to the competent authority. This may include HTA dossiers and requests for pricing and/or reimbursement.
Horizon Scanning	Systematic identification of upcoming medicinal products, typically conducted by public authorities to anticipate budget impact and clinical value and to allow for resource planning and product selection for HTA.
HTA Selection	Authorities select or decide whether a product undergoes an HTA process.
Maximum Price Application	Application by the HTD for a maximum price.
Pre-Submission Activity	Pre-Submission Activity refers to formal or routinely required interactions and procedural steps undertaken by an HTD with national authorities prior to dossier submission. This may include country-specific administrative prerequisites such as notifications of intent or pre-submission meetings where these form an established part of the standard national pathway. Optional early advice or consultation activities were not included unless they were routinely embedded in the standard process.
Price Decision	Formal determination of the price by the responsible authority. In cases where two distinct price decisions were made by different authorities, both steps were retained to reflect the structure of the process.
Price Negotiation	Negotiations between the HTD and the relevant authority (e.g., payer or ministry) to agree on the final reimbursed price and/or financial conditions, including managed entry agreements where applicable.
Recommendation	Recommendation steps were included when a formal advisory opinion was issued by an entity distinct from the body responsible for the final reimbursement decision.
Reimbursed Access	Medicinal product becomes available to patients under reimbursement conditions. Publication of pricing and reimbursement decisions (e.g., official gazette notifications or inclusion in national reimbursement lists) was not defined as a separate process step. Instead, it was considered an integral part of the “Reimbursed Access” step, as it typically represents the formal legal or administrative activation of reimbursement decisions.
Reimbursement Decision	Final determination by the competent authority on whether the medicinal product is eligible for reimbursement within the public healthcare system, including positive, conditional, or negative outcomes.

Abbreviations: HTA: health technology assessment; HTD: health technology developer.

## Data Availability

The original contributions presented in this study are included in the article/Supplementary Material. Further inquiries can be directed to the corresponding author.

## Supplementary Materials

The following supporting information can be downloaded at: https://www.mdpi.com/article/10.3390/jmahp14030055/s1, Figure S1: Average time to market access based on EFPIA Patients W.A.I.T. Indicator 2025 Survey and sequence plots of process steps for in- and outpatient pathways; Figure S2: Cluster selection for in- and outpatient pathways; Figure S3: Silhouette plots for in- and outpatient pathways; Figure S4: Boxplots displaying the distribution of medicinal product availability and time to access based on EFPIA Patients W.A.I.T. Indicator 2025 Survey; Figure S5: Boxplots displaying the distribution of GDP per capita and health expenditure per capita based on eurostat data; Table S1: Statistic results for between cluster comparison with Kruskal-Wallis rank sum tests.
