# Supplementary material for: The Post-Authorisation Maze: Mapping of European Pharmaceutical Market Access Pathways in the Early JCA Era
Source: J Mark Access Health Policy. 2026 Sep 17;14(3):55. doi: 10.3390/jmahp14030055 (PMC13608677; doi:10.3390/jmahp14030055)
Supplement: Supplementary file 1 [file jmahp-14-00055-s001.zip › jmahp-4515296-supplementary.pdf]

The Post-Authorisation Maze: Mapping of European Pharmaceutical Market Access Pathways in the Early JCA Era

Supplementary Material

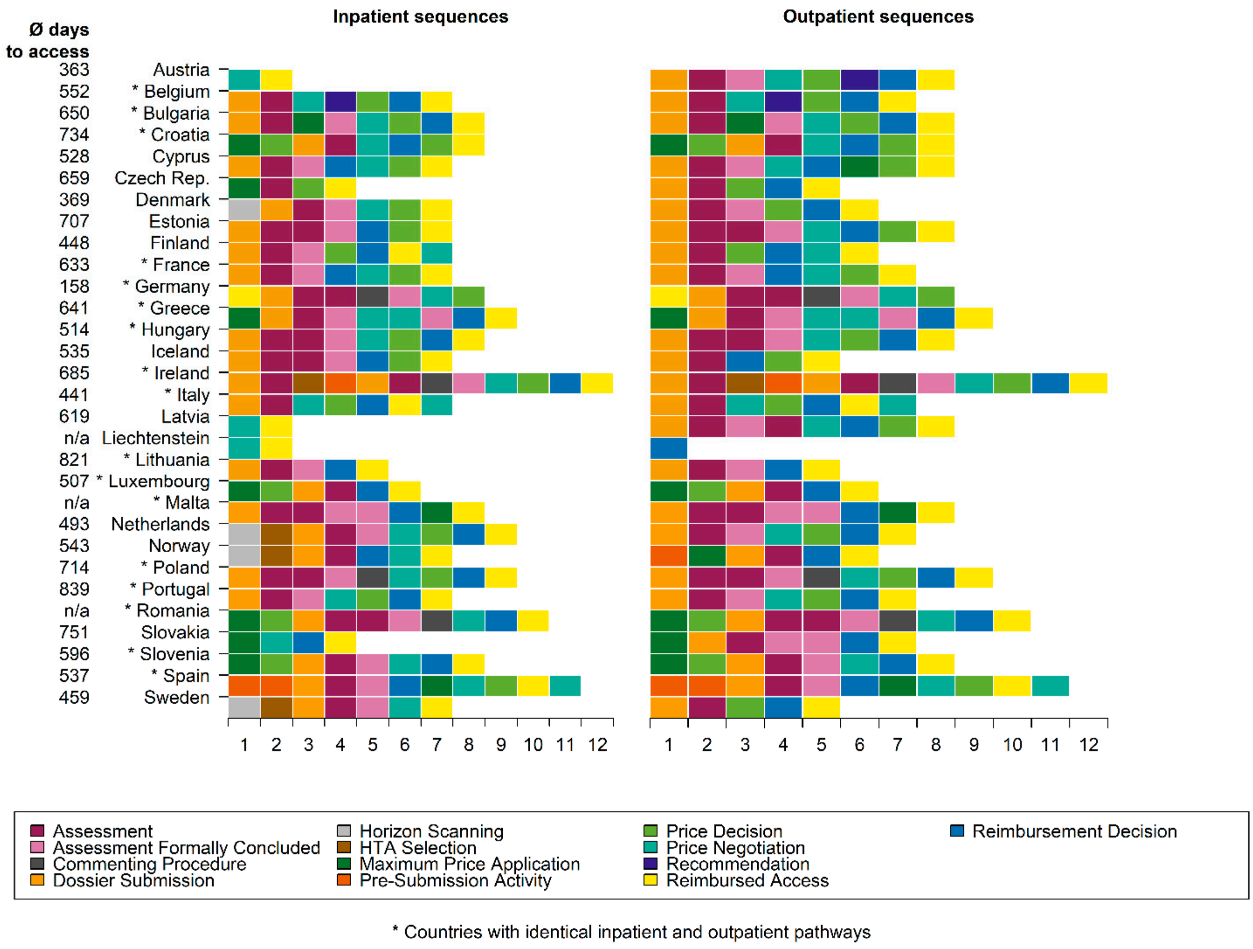

Supplementary Figure S1: Average time to market access (days) based on EFPIA Patients W.A.I.T. Indicator 2025 Survey [1] (left column) and sequence plots of process steps for inpatient (left sequence plots) and outpatient pathways (right sequence plots), ordered alphabetically from top to bottom.

.

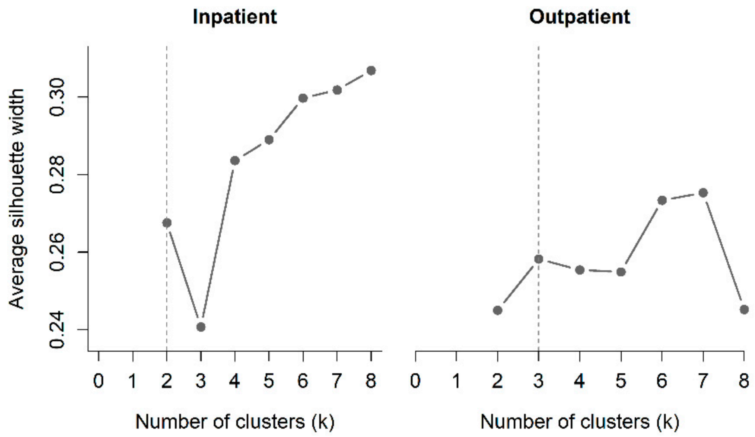

Supplementary Figure S2. . **Cluster selection** for inpatient (left column) and outpatient pathways (right column). Panels show silhouette-based selection of the number of clusters (k), with the chosen solution marked by the dashed line.

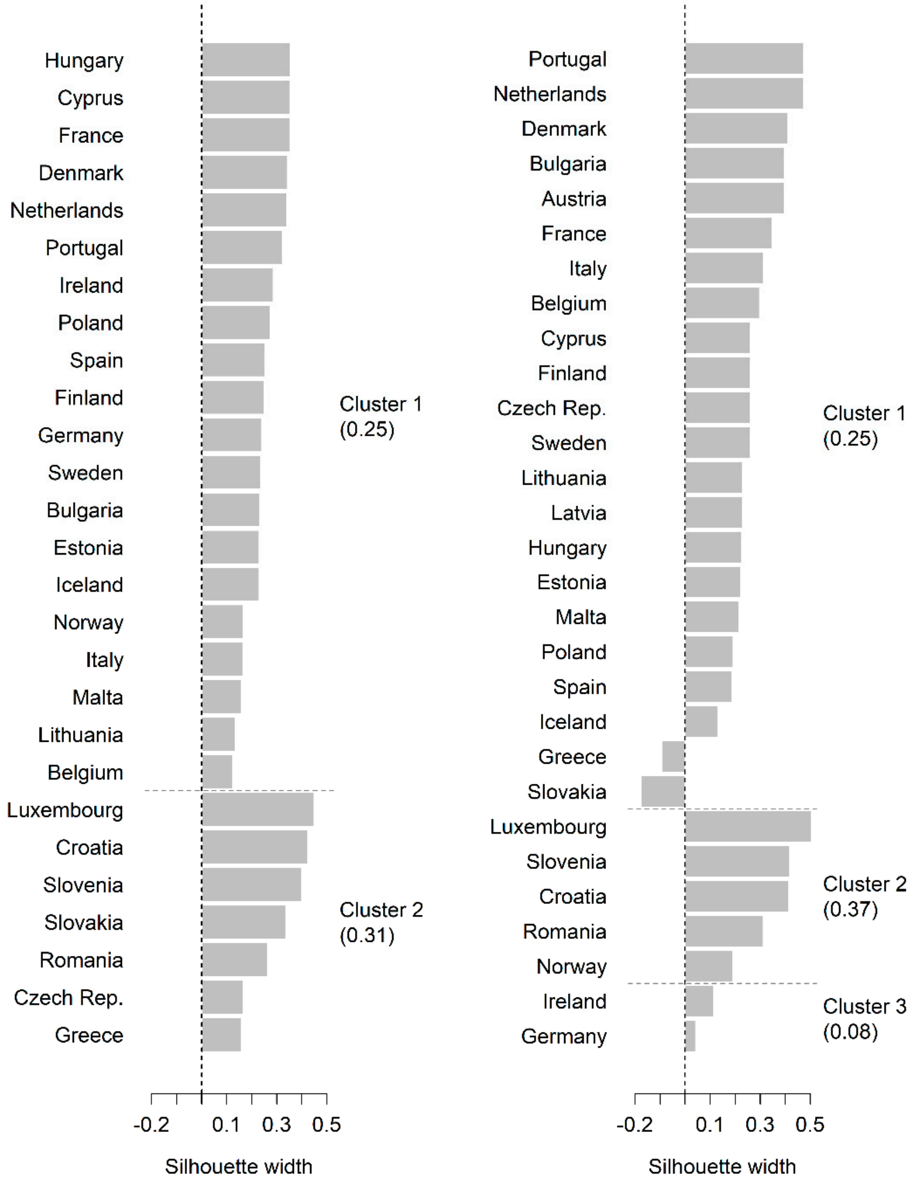

Supplementary Figure S3: **Silhouette plots** for inpatient (left column) and outpatient pathways (right column). Panels show silhouette plots of the final clustering solutions, with cluster-specific average silhouette widths reported in brackets.

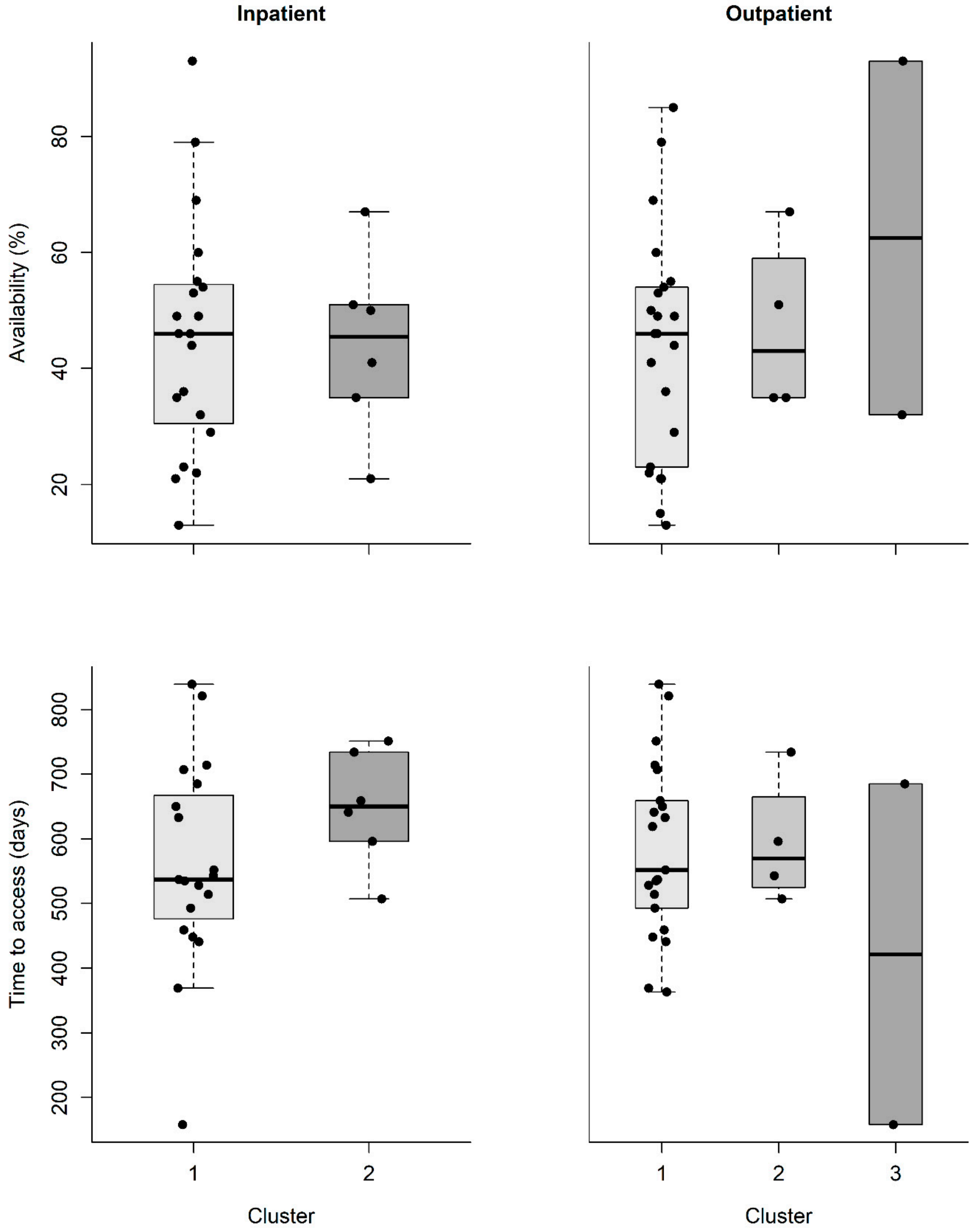

Supplementary Figure S4 :Boxplots display the distribution of medicinal product availability (%; upper panel) and time to access (days; lower panel) based on EFPIA Patients W.A.I.T. Indicator 2025 Survey [1], across clusters identified in the sequence analysis, separately for inpatient (left panels) and outpatient pathways (right panels). Boxes represent the interquartile range (IQR), with the horizontal line indicating the median. Whiskers extend to the most extreme values within 1.5×IQR. Individual observations are overlaid as jittered points.

Note. Countries with missing values in the EFPIA Patients W.A.I.T. Indicator 2025 Survey were omitted from the analysis. Due to the low number of countries per cluster these data should be interpreted with caution.

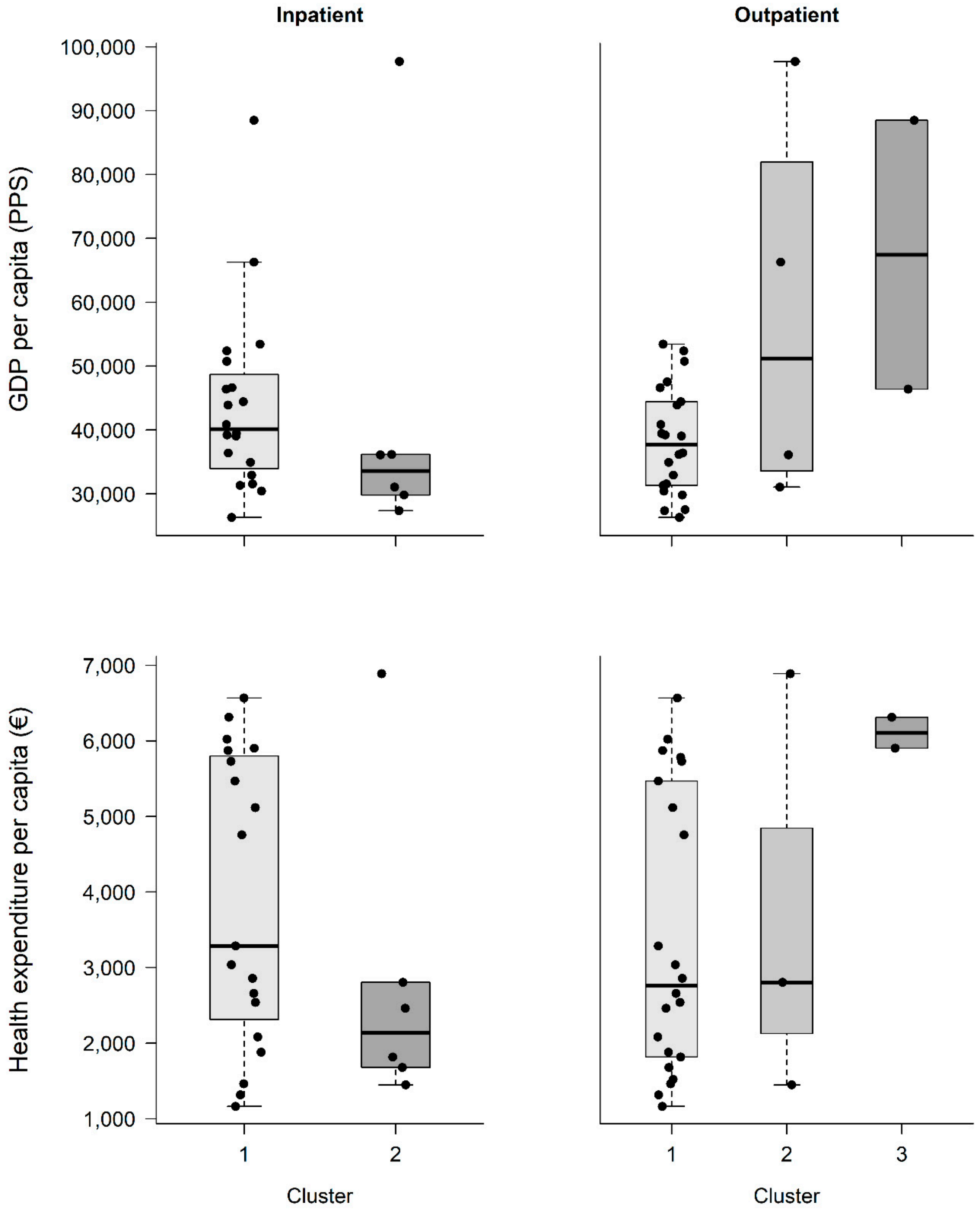

Supplementary Figure S5: Boxplots display the distribution of GDP per capita (Purchasing Power Standard [PPS], upper panel) and health expenditure per capita (€, lower panel) based on eurostat data [2, 3], across clusters identified in the sequence analysis, shown separately for inpatient (left panels) and outpatient (right panels) pathways. Boxes represent the interquartile range (IQR), with the horizontal line indicating the median. Whiskers extend to the most extreme values within 1.5×IQR. Individual observations are overlaid as jittered points.

Note. Countries with missing values in the Eurostat data sets were omitted from the analysis. Due to the low number of countries per cluster these data should be interpreted with caution.

	Inpatient results				Outpatient results			
**Variable**	χ^2	*n*	*df*	*p*	χ^2	*n*	*df*	*p*
**Pathway length**	0.120	27	1	0.729	2.879	29	2	0.237
**Percentage of availability**	0.004	26	1	0.951	0.503	28	2	0.778
**Time to access**	1.96	25	1	0.162	0.352	27	2	0.839
**HEP**	1.312	25	1	0.252	3.498	27	2	0.174
**PPS2024**	2.133	26	1	0.144	3.664	28	2	0.160

Supplementary Table S1: Statistic results for between cluster comparison with Kruskal-Wallis rank sum tests. Analyses were conducted separately for in- and outpatient clusters.

Supplementary References

1. IQVIA. EFPIA Patients W.A.I.T. Indicator 2025 Survey. 2026. Accessed: 15.07.2026. Available from: https://www.efpia.eu/media/mnfdwzax/efpia-patients-wait-indicator-2025.pdf
2. Eurostat. Gross domestic product (GDP) and main components per capita - annual data: gross domestic product at market prices, current prices, purchasing power standard (PPS, EU27 from 2020) per capita, 2024 [nama_10_pc]. 2026.
3. Eurostat. Health care expenditure by financing scheme: all financing schemes, euro per inhabitant, annual data [hlth_sha11_hf]. 2026.
